# Why Nurses Resign: A Qualitative Study Across Life Stages and Implications for Workforce Management

**DOI:** 10.1155/jonm/3278481

**Published:** 2026-08-31

**Authors:** Elisabetta Cesaro, Lucia Stivanello, Elisa Daniele, Elena Massarotto, Michela Zanella, Nicola Trevisan, Fabio Perina, Mario Degan

**Affiliations:** ^1^ Department of Medicine, University of Padua, Padua, Italy, unipd.it; ^2^ Healthcare Profession Department, Padua University Hospital, Padua, Italy, unipd.it; ^3^ Human Resources Unit, Padua University Hospital, Padua, Italy, unipd.it; ^4^ Hospital Management Unit, Padua University Hospital, Padua, Italy, unipd.it

## Abstract

**Background:**

Voluntary resignation among nurses poses a growing challenge for healthcare organisations, with implications for workforce stability and quality of care. While intention to leave has been widely examined, little is known about the experiences of nurses who have already enacted their decision to resign, particularly across different life stages.

**Aim:**

To explore the reasons underlying voluntary resignation among nurses and examine how these reasons vary across generational cohorts, with the goal of informing life‐stage‐sensitive workforce retention strategies.

**Methods:**

A qualitative descriptive study was conducted in a large Italian university hospital. Nurses who voluntarily resigned between March and November 2025 participated in semistructured exit interviews. Data were collected through detailed field notes and analysed inductively using thematic analysis.

**Results:**

Sixty‐six nurses participated. Resignation emerged as a multifactorial process shaped by the interaction between organisational conditions and evolving life‐stage priorities. Generation Z participants reported insufficient onboarding, unmet expectations and difficulties balancing work with academic or personal commitments. Generation Y emphasised workload pressures, limited recognition, poor organisational climate and restricted career development. Generation X highlighted physical strain, scheduling needs and logistical barriers such as commuting and parking difficulties. Across all groups, chronic understaffing, limited flexibility and insufficient managerial support were consistently identified as key contributors.

**Conclusions:**

Voluntary resignation reflects a complex interplay between organisational factors and life‐stage‐specific needs rather than isolated dissatisfaction. The findings highlight under‐recognised determinants—such as micrologistical stressors and personal development aspirations—that influence enacted turnover.

**Implications for Nursing Management:**

Nurse managers should adopt life‐stage‐sensitive retention strategies, including structured onboarding, supportive leadership, flexible scheduling, recognition of competencies and attention to logistical and quality‐of‐life factors. Addressing both organisational and relational dimensions may enhance staff retention and contribute to a more sustainable workforce.

**Summary**
• What is known about this topic
◦ Voluntary resignation among healthcare workers is increasing globally and threatens workforce stability and quality of care.
◦ Most existing studies focus on intention to leave rather than on the experiences of nurses who have already resigned.
◦ Work–life imbalance, workload pressures and limited managerial support are recognised contributors to turnover.
◦ Little is known about how resignation drivers differ across generational cohorts and life stages.
• What this paper adds
◦ Provides one of the few qualitative analyses of enacted voluntary resignation among nurses, moving beyond intention‐to‐leave research and capturing retrospective accounts of completed turnover decisions.
◦ Identifies how resignation drivers differ across generations and life stages, highlighting the interaction between organisational conditions and evolving professional and personal priorities.
◦ Reveals under‐recognised determinants of turnover, including micrologistical stressors such as commuting burden and parking difficulties.
◦ Demonstrates that relational and identity‐related factors—such as feeling valued, recognised and supported—play a critical role in resignation decisions.
◦ Offers actionable, life‐stage‐sensitive implications for nursing management to strengthen workforce retention.

## 1. Introduction

The demand for health services is rising, driven by an ageing population with increasingly complex healthcare needs. As life expectancy rises, the prevalence of long‐term conditions is expected to grow, with more individuals managing multiple chronic illnesses simultaneously [1]. In this context healthcare organisations worldwide are facing increasing challenges in retaining their workforce, particularly among frontline nurses.

Although intention to leave (ITL) the profession has been extensively studied—especially among nurses—far less is known about the experiences of nurses who have already enacted their decision to resign. This gap limits the ability of nurse managers to design effective, evidence‐based retention strategies. Among nurses, the average ITL in the profession is estimated to be 30% in Italy [2] and 22.8% among intensive care nurses in Europe [3]; 43.5% instead expressed the intention to stay in the profession [4]; physicians, on the other hand, have an overall ITL the hospital of 16.5% [5]. Therefore, Italy shows a lower nurse‐to‐physician ratio, driven by a high physician density and a historical shortage of nurses [6].

Maintaining a balance between personal life and professional responsibilities is often challenging, as is the lack of professional recognition and the need for greater autonomy [7, 8]. Competency in the role has also been linked to the ITL [9]. Dabaghi et al. identified four main antecedents of ITL: demographic, psychological, interpersonal and occupational factors [10]; furthermore, higher ITL in hospitals has been observed among younger nurses, those who served on the frontline against COVID‐19, and individuals experiencing lack of equipment, emotional exhaustion, dissatisfaction with work prospects, or dissatisfaction with the use of professional skills [11, 12].

A ward transfer may offer a viable option prior to considering resignation [13]; among nurses, transfer requests are predominantly of undergraduates with a mean age of 30.8 ± 6.4 years and an average work experience of 4.6 years. The emergency department and intensive care unit were the most common departments from which transfer requests originated, primarily due to health‐related issues, long shifts and the desire for professional advancement [14].

Such ITL, the profession or request for ward/hospital transfers, contributes to high turnover in care settings, which in turn may affect the quality of nursing care [15]. Understanding how these dynamics unfold among nurses, healthcare assistants and technicians is crucial to develop interventions that support staff retention, reduce turnover and safeguard care quality. Moreover, little is known about how resignation drivers vary across life stages. Generational differences in work values, expectations and personal responsibilities may shape how nurses perceive organisational demands and decide to leave [16]. However, the explanatory value of generational categories remains debated. Recent scholarship argues that many apparent generational differences may instead reflect age, career stage, or lifespan developmental processes and recommends adopting more nuanced life‐span perspectives rather than treating generations as fixed explanatory constructs [17]. Although life stages and generations are likely to overlap, they are neither the same construct nor the same variable in operational terms [18]. Empirical evidence comparing resignation experiences across generational cohorts and life stages remains limited, particularly within European healthcare systems. This gap is relevant for understanding why different generations and nurses at different life stages decide to resign, which is useful for the future development and implementation of tailored retention policies. The healthcare workforce is highly heterogeneous; for example, older nurses (> 55 years) represent approximately 40% of the workforce [19]. Consequently, no ‘one‐size‐fits‐all’ approach is likely to be effective for staff retention [20].

While managers and supervisors have limited scope to influence resignations driven by personal reasons, they can implement organisational changes to improve retention when employees leave because of working conditions or organisational culture [21].

Despite increasing concerns about healthcare workforce shortages, evidence based on actual resignation experiences rather than turnover intentions remains scarce, particularly in European healthcare settings.

Unlike intention‐to‐leave studies, research on enacted resignation suggests a shift from anticipated withdrawal to actual decision‐making at the point where organisational dissatisfaction translates into action.

This study addresses these gaps by exploring the reasons underlying voluntary resignation among nurses in a large Italian university hospital, examining how these reasons differ across generational groups and life stages, and identifying actionable strategies to support workforce retention.

This study addressed the following research questions: (a) What organisational and personal factors lead nurses to enact voluntary resignation? (b) How do these factors differ across life stages? (c) Which determinants emerge only after resignation has been enacted?

## 2. Method

### 2.1. Design

We adopted a qualitative descriptive approach within an interpretivist paradigm, aiming to explore how participants made sense of their resignation experiences. An inductive thematic analysis was conducted following Braun and Clarke’s approach [22], allowing themes to emerge from the data without imposing pre‐existing theoretical frameworks. To ensure methodological rigour, the study adhered to the guidelines in the Consolidated Criteria for Reporting Qualitative Research (COREQ) [23]. While the initial coding and theme development were conducted inductively, generational theory was used as a sensitising lens in interpretation of patterns across participants. According to generational theory, individuals born within the same historical timeframe may share values and perspectives shaped by common sociocultural experiences [24]. In this study, generational cohorts were not treated as fixed or homogeneous categories but rather as heuristic devices to explore potential differences in how participants understood and narrated their work‐related decisions and their life stage needs.

### 2.2. Study Setting and Recruitment

The study was conducted at a University Hospital in Italy, a public facility with 1682 beds and nearly 7000 total employees. The hospital is a regional hub.

Data were collected between March and November 2025. The study period was defined a priori to capture all nurses who submitted a voluntary resignation within a consecutive cohort at the study hospital. This timeframe aligned with organisational access to resignation records and allowed inclusion of all eligible cases during the recruitment window, ensuring full coverage of the organisational population of interest and avoiding sampling bias linked to selective recruitment. Participation was voluntary, and no incentives were offered.

All eligible nurses were invited to participate in a face‐to‐face exit interview via institutional email. The relatively large sample size reflected the broad recruitment frame and the intention to capture a wide range of perspectives across different clinical contexts within a single institution, rather than to achieve statistical representativeness. Sample adequacy was evaluated throughout the study through regular discussions within the research team, taking into account the depth, recurrence and diversity of participants’ accounts in relation to the study aim [25].

### 2.3. Inclusion and Exclusion Criteria

Eligibility was defined as all nurses who voluntarily resigned from the hospital within the study period (March–November 2025). Inclusion criteria were (a) voluntary resignation submitted within the study period; (b) employment as a nurse; and (c) voluntary participation. Exclusion criteria were employment under a fixed‐term contract.

### 2.4. Data Collection

Exit interviews were conducted from March to November 2025; the interview was carried out by a female health professions manager and the researcher (EC), a female PhD student with experience in qualitative research; the researcher has no relationship established prior to study commencement. Semistructured face‐to‐face interviews were conducted with each participant. In accordance with participants’ preferences and ethical considerations, no audio or video recordings were made. Instead, the interviewer and the researcher took detailed field notes during each interview and expanded and finalised these notes immediately afterwards to produce a comprehensive written account of the conversation. Whenever possible, key points were restated to participants during the interview to confirm understanding. This approach is recognised within qualitative research when recording is not feasible, although it may limit the richness of verbatim quotations. Having two interviewers present during each interview enhanced the richness of the data by enabling the documentation of pauses, nonverbal behaviours, contextual elements and other subtle interactional nuances alongside participants’ verbal responses. By interviewing nurses after they had voluntarily resigned, this study captures retrospective accounts of completed turnover decisions. As participants were no longer employed by the organisation, they may have felt more comfortable discussing sensitive organisational issues and reflecting openly on the factors that influenced their decision. The qualitative approach enables an in‐depth exploration of resignation experiences, capturing the complexity and context‐specific factors that quantitative studies are often unable to identify.

Each interview lasted approximately 25 min on average and was conducted in the health profession department meeting room. The extended timeframe did not reflect slow recruitment but rather the episodic nature of resignations: resignations occurred intermittently, in small waves, with no predictable pattern. Consequently, interviews were conducted whenever new resignations occurred and participants became available. Given the qualitative descriptive design and the defined organisational population, the aim was to achieve informational adequacy rather than statistical representation. Data collection continued throughout the study period until sufficient depth and richness of data had been achieved, with no substantially new themes emerging toward the end of the recruitment window.

To explore the phenomenon, the interview guide consisted of two parts: (1) sociodemographic information collecting information on (a) age (years); (b) overall professional experience (years); (c) hospital tenure (years) and (2) open‐ended questions designed to explore (a) the underlying causes of voluntary resignation; (b) respondents’ future intentions; (c) suggestions for improving nurses’ retention (Supporting 2). The interview guide was developed by the researcher in collaboration with HR managers and healthcare professions managers, based on a review of the relevant literature. The guide was subsequently piloted in two interviews, which were not included in the final analysis. Following this pilot phase, the interview questions were refined to improve clarity and relevance before being used in the main study.

### 2.5. Data Analysis

The expanded field notes were imported into Atlas.ti software for systematic management of the textual corpus. Data analysis followed an inductive thematic analysis approach, in line with the six‐phase procedure proposed by Braun and Clarke [22]: familiarisation with the data through repeated reading of the transcripts, generation of initial codes by identifying meaningful segments of text, searching for themes by grouping codes into coherent conceptual clusters, reviewing and refining themes through the construction of a thematic map, defining and naming themes, clarifying their essence and their relationship to the research objectives, producing the final report, which synthesised the main themes and subthemes with illustrative quotations. Data analysis was conducted collaboratively by two members of the research team. An initial subset of transcripts was independently coded by both researchers to enhance analytic rigour. Coding decisions were subsequently compared and discussed in depth, and discrepancies were resolved through consensus. This iterative process informed the development and refinement of the coding framework.

### 2.6. Ethical Considerations

The transcribed interview files were anonymised using progressive alphanumeric codes (e.g., P1, P2) and stored in a secure digital database accessible only to members of the research team.

The study has been approved by the Local Ethic Committee (CET‐ACEV) with protocol number 10658 on 11/02/2026.

### 2.7. Rigour and Reflexivity

Data collection occurred within the organisational context of formal exit interviews. As these conversations were conducted during the resignation process and involved a health professions manager, audio recording was not feasible due to institutional norms and participant preference. This context may have influenced the depth of disclosure and introduced potential social desirability bias.

To mitigate this risk, the research team employed several strategies: (a) dual field‐note taking to enhance completeness and accuracy; (b) immediate expansion of notes after each interview; (c) member clarification during interviews; (d) consistent use of a semistructured guide; and (e) investigator triangulation through independent coding and team discussions. Attention was paid to ensuring that participants felt free to express their views, and efforts were made to minimise any potential influence on participants’ responses.

The qualitative descriptive design, which prioritises a low‐inference interpretation of participants’ accounts, was considered appropriate for this context, as the aim was to capture reported reasons for resignation rather than to generate deep interpretative theory. Nevertheless, the absence of verbatim transcripts may have limited the granularity of linguistic analysis and the richness of illustrative quotations.

Reflexive attention was given to the positionality of the research team. The involvement of a managerial figure may have shaped participants’ narratives, potentially leading to moderated expressions of dissatisfaction. The research team remained critically aware of this dynamic during analysis and interpreted findings within the constraints of the organisational setting. Any discrepancies were resolved through iterative discussion until full consensus was achieved. The final themes, subthemes and codes were reviewed and refined through discussion with the broader research team, allowing for consensus to be reached. This collaborative process contributed to the credibility and trustworthiness of the analysis by supporting investigator triangulation.

### 2.8. Findings

A total of 66 nurses participated in the study (Supporting Table 1). For each participant, information was additionally recorded on the total duration of employment at the hospital (Table 1).

**TABLE 1:** Nurses’ professional data.

Nurses	Mean age (SD) (years)	Mean hospital tenure (SD) (years)
Nurses	32.5 (±8.1)	6.23 (±8.1)

*Note:* Nurses were divided into three generational cohorts: *n*. 22 (33%) Generation Z (born after 1997) nurses, *n*. 38 (58%) Generation Y (born between the years 1981 and 1996), *n*. 6 (9%) and Generation X (born between the years 1965 and 1980). 22 were male nurses, and 44 were female nurses.

From the analysis among different generation nurses, 6 themes, 12 subthemes and 25 codes were identified and divided into generations (attached in Table 2).

**TABLE 2:** Description of the coding tree.

Themes	Subthemes	Codes	Quotations
Negotiating life priorities and work–life misalignment	Personal and financial drivers of relocation	Moving closer to home	‘I need to move closer to my family’. (P8)
		High rental costs	‘Rents here are too high for my salary’ (P33)
	Work–life incompatibility	Study–work conflict	‘Work cannot be reconciled with my study commitments’. (P48)
		Early emotional exhaustion	‘I’m already emotionally drained’.(P70)

Early‐career transition challenges and unmet support needs	Lack of organisational support	Insufficient onboarding	‘I was given autonomy too quickly, without adequate guidance’. (P44)
		Feeling abandoned	‘They left me alone with all the patients with no feedback’ (P61)
		Premature autonomy	‘They gave me autonomy very quickly’. (P44)
	Unsustainable workload intensity	Excessive workload	‘The workload is huge and responsibilities are very high’. (P83)
		Staff shortages	‘We are always understaffed’. (P18)

Experiencing work–life strain within challenging workplace environments	Personal life incompatibility	Work–family conflict	‘Work is not compatible with my family situation’. (P62)
		Excessive workload and chronic fatigue	‘The workload is huge… I don’t want to be treated like a number’. (P84) ‘I’m exhausted; emotional stress is affecting my health’. (P51)
	Dysfunctional workplace relationships	Conflicts	‘This is a toxic environment’. (P29)
		Lack of listening	‘Nobody really listens to our problems’. (P16)

Experiencing limited recognition and constrained professional growth	Blocked professional development	No continuing professional development	‘I asked several times for courses, but they were denied’. (P46)
		Desire for internal mobility	‘I would have liked to experience other wards’. (P84)
	Lack of professional recognition	Skills not valued	‘I was never formally recognised for my advanced skills’. (P51)
		Unrecognised competencies	‘I had many responsibilities, but no recognition’. (P59)

Accumulating physical strain and everyday workplace burdens	Physical demands and age‐related fatigue	Chronic fatigue	‘Physically I can’t do it anymore’. (P81)
		Reduced recovery capacity	‘At my age, recovery after a night shift is difficult’. (P39)
	Cumulative stress exposure	Parking difficulties	‘I can’t find parking’. (P81)
		Emotional exhaustion	‘I’m 59 and exhausted… stress has built up over time’. (P41)

Negotiating work–life boundaries	Caregiving as a catalyst for resignation	Need for more personal time	‘I need to work part‐time to manage my family responsibilities’. (P34)
	Reorientation toward sustainable work	Denied part‐time	‘I asked for part‐time but it was never granted’. (P61)
		Preference for day shifts	‘Day shifts are the only sustainable option for me’. (P34)

Patterns across generational cohorts were interpreted as reflecting underlying life‐stage differences rather than stable generational effects. The generational framework was used as a sensitising lens for analysis, while shared experiences across cohorts were also retained and reported when analytically relevant.

### 2.9. Generation Z

#### 2.9.1. Theme 1—Negotiating Life Priorities and Work–Life Misalignment

The interviewed nurses reported resigning primarily due to the distance from their families and places of origin. As one participant stated, ‘*I want to be closer to my parents’* (P49). Family proximity strongly influences their sense of well‐being and has become a priority for achieving work–life balance. They experience stress due to being far from home and relatives and express a desire for stability. Nurses relocating from other cities or regions also reported additional stressors related to housing difficulties and the high cost of living. Younger nurses, furthermore, experience an emotional exhaustion due to a missed work–life balance. ‘*I feel overwhelmed; my health has been affected, with migraines and other symptoms’.* (P79).

Nurses, in fact, highlighted the need for predictable working hours and schedules that can be reconciled with other life commitments, such as attending university courses or having a social life. As one participant noted, ‘*Work cannot be reconciled with my study commitments*’ (P48). Participants described a clear incompatibility between work schedules and ongoing academic commitments.

#### 2.9.2. Theme 2—Early Career Transition Challenges and Unmet Support Needs

New employees reported insufficient onboarding support and mentoring. Generation Z nurses also reported a lack of support and insufficient shadowing, which often led them to work independently before feeling fully prepared. The brief onboarding to an unfamiliar hospital ward made them feel vulnerable when performing demanding duties and exposed them to high responsibilities. ‘*They gave me autonomy very quickly*’. (P44). Nurses also noted that, in some cases, their ward preferences expressed during the job interview were not accommodated, and in consequence, they do not like their working area. They report an early emotional exhaustion connected also with their perception of excessive workload, responsibility and staff shortages ‘…*we are always understaffed*…’ (P18).

### 2.10. Generation Y

#### 2.10.1. Theme 3—Experiencing Work–Life Strain Within Challenging Workplace Environments

Nurses reported feeling unsafe and extremely fatigued due to excessive workloads, inadequate nurses‐patient ratios, and frequent requests to return from rest days to cover staffing gaps. These conditions contribute to symptoms of burnout and a general sense of discomfort within the hospital environment. Participants also highlighted chronic understaffing, particularly during night shifts, which they feel is heavier than daytime.

Resigning in order to work closer to home appeared closely linked to family responsibilities.

The workplace climate and interprofessional relationships emerged as critical factors influencing the experiences of nurses. Participants reported a generally stressful organisational climate characterised by interpersonal conflicts, poor communication, low team cohesion and insufficient emotional support, which contributed to a perception of a toxic work environment. As one nurse expressed, ‘*We spend so much time here, yet we feel poorly supported’* (P36). Challenges were also evident in interactions with medical staff. Several participants highlighted poor nurse–physician collaboration, hierarchical communication, limited respect from physicians and a lack of shared decision‐making. ‘*There are people in the team who create difficulties’.* (P84). Nurses frequently described being treated as functionaries rather than active contributors to patient care, as illustrated by the statement, ‘*Physicians treat you as a mere executor’* (P6). These findings suggest that both the overall team climate and the quality of interprofessional relations significantly affect staff satisfaction, engagement and retention.

#### 2.10.2. Theme 4—Experiencing Limited Recognition and Constrained Professional Growth

One of the factors contributing to staff turnover is the perceived lack of opportunities for professional growth within the hospital setting, despite specialisation courses or prior work experience. In contrast, in their perception, private healthcare settings often offer faster career progression.

Nurses also express a desire to enhance their skills, although not valued, through courses and conferences, which are frequently inaccessible due to staffing shortages. Nurses report insufficient development support and mentoring, with one stating, ‘I would have liked more training opportunities’ (P59). This perception is further reinforced by feelings of professional insufficient recognition, as exemplified by the statement, ‘I did not feel professionally valued’ *(P51) and* ‘There has been little recognition for everything I have done’. (P58), reflecting both a lack of formal recognition of competencies and inadequate remuneration.

Participants also described limited managerial presence and insufficient organisational support, which contributed to feelings of vulnerability and dissatisfaction.

### 2.11. Generation X

#### 2.11.1. Theme 5—Accumulating Physical Strain and Everyday Workplace Burdens

Generation X nurses described their work as increasingly physically demanding, often linking their decision to resign to a sense of age‐related fatigue and reduced physical resilience. As one participant noted, ‘*The workload is too heavy’* (P87), highlighting how routine tasks, high patient acuity and demanding shifts—particularly night duties—contributed to cumulative physical exhaustion. Several participants reported that, with age, recovering from night shifts became progressively more difficult, exacerbating feelings of strain and diminishing their overall well‐being.

In addition to the physical demands of clinical work, nurses repeatedly emphasised the impact of logistical stressors on their daily experience. The lack of available parking was one of the most frequently cited sources of frustration: ‘*I arrive already stressed because I can’t find parking’* (P41). Participants described these structural barriers—such as long commuting times, limited transport options and chronic parking shortages—as contributing to a sense of ‘*starting the shift already worn out’*. (P26) These micrologistical obstacles, although not directly clinical, were perceived as significant amplifiers of daily stress, adding to time pressure, emotional burden and overall dissatisfaction with the work environment.

#### 2.11.2. Theme 6—Negotiating Work–Life Boundaries

Several participants explained that, after many years of service, they sought ‘*more regularity and enough energy to care for my family after work’*. (P66) The rigid scheduling patterns of hospital settings, combined with unexpected shift changes and requests to cover staffing shortages, were described as incompatible with the needs of older nurses who often juggle caregiving responsibilities for children, elderly parents, or both.

Chronic understaffing—particularly during night shifts—further intensified this imbalance. ‘*There is a chronic shortage of staff’.* (P41). Participants reported that reduced staffing led to heavier workloads and increased physical and emotional strain, reinforcing their perception that the hospital environment could no longer accommodate their evolving personal and family needs.

For many Generation X nurses, achieving a satisfactory work–life balance, also associated with proximity to home,became a central factor in their decision to resign. Participants highlighted the importance of shift organisation, noting a growing preference for day shifts and reduced tolerance for frequent night duties. They expressed a strong desire for predictable and regular schedules that would allow them to manage personal and family responsibilities more effectively.

### 2.12. Nurses’ Feedback for Improvement

The suggestions provided by resigning staff offer important insights into areas where organisational practices could be strengthened. Participants commonly recommended increasing staffing levels and ensuring more sustainable workloads, particularly during night shifts and high‐acuity periods, to safeguard both staff wellbeing and patient safety. Several highlighted the need for more structured onboarding pathways and ongoing access to meaningful training opportunities, noting that advanced skills and prior experience should be better recognised and rewarded. Suggestions also emphasised improving communication and leadership practices, fostering a more supportive and collaborative climate, and ensuring that employee concerns are acknowledged and addressed in a timely manner. Practical recommendations—such as expanding parking availability, enhancing canteen services and offering housing or logistical support for nonlocal employees—were also recurrent. Collectively, these suggestions underline the importance of addressing organisational, relational and structural factors to improve the working environment and potentially mitigate future turnover.

## 3. Discussion

This study explored the reasons underlying voluntary resignation among nurses in a large Italian university hospital, moving beyond the widely studied concept of ITL to examine the experiences of individuals who had already enacted their decision to resign.

The mean age of resigning nurses (32.5 years) and their average hospital tenure (6.2 years) indicate that turnover in this context predominantly affected early‐ and midcareer professionals, consistent with previous research reporting higher ITL among younger nurses and those with limited work–life balance [11, 14, 26, 27]. These findings reinforce the need for retention strategies tailored to life‐stage priorities.

Across all generational cohorts, work–life imbalance emerged as the primary driver of resignation. This aligns with extensive evidence linking work–life conflict to turnover intentions among nurses [28].

In particular, work–life tensions, organisational constraints (such as staffing shortages and workload intensity) and perceived limitations in support and recognition emerged as recurring and interrelated features of participants’ accounts. Rather than functioning as discrete categories, these elements interact to produce a cumulative and relational experience of strain that is embedded within both workplace structures and professional trajectories. This is consistent with previous studies linking high workload to turnover intentions [29–32]. Participants also highlighted the importance of leadership quality, communication and emotional support—factors widely recognised as influencing job satisfaction and intention to stay [8, 30, 33, 34]. In particular, younger nurses reported insufficient mentorship and rapid exposure to responsibilities, echoing evidence that structured onboarding improves competency and job satisfaction [35].

These patterns do not represent universal or deterministic explanations, but rather interpretive threads that cut across generations and life stages, highlighting the shared situatedness of nurses within similar organisational environments.

When this cumulative strain is further reinforced by contextual quality‐of‐life factors—such as commuting distance, parking difficulties, or housing challenges—it may reach a threshold beyond which resignation becomes the preferred and enacted solution. In this sense, voluntary resignation emerged not as the consequence of a single adverse event but as the outcome of an evolving interaction between organisational conditions and individual life‐stage needs. Our findings can be interpreted through the lens of the Job Demands–Resources (JD–R) model, whereby organisational constraints such as workload, staffing shortages and limited managerial support resemble job demands, while recognition, leadership and supportive relationships function as job resources [36]. However, our findings also suggest that the impact of these organisational factors depends on nurses’ life‐stage priorities, indicating that a life course perspective complements the JD–R model by explaining why similar working conditions may lead to different resignation decisions across individuals.

This interpretation extends existing turnover models by suggesting that resignation represents the outcome of a cumulative process rather than the direct consequence of isolated organisational stressors (Supporting Figure 1).

While these organisational factors were shared across participants, their meaning and impact varied according to nurses’ life stages and personal circumstances.

Generational and life stage differences shaped how nurses experienced work–life imbalance. Younger nurses (Generation Z) reported difficulties reconciling work with academic commitments, insufficient onboarding and early emotional exhaustion. These findings are consistent with evidence that Generation Z values mental health, flexibility and continuous feedback [37, 38]. In contrast, Generation Y participants highlighted chronic workload pressures, limited career development and adverse organisational climates. Previous studies similarly report higher turnover intentions among Generation Y compared with Generation X [39], although such differences may reflect life‐course dynamics rather than stable generational traits. Our findings support this interpretation: Midcareer nurses often faced family responsibilities and sought greater stability, echoing evidence that work–family conflict is particularly salient in this group [40, 41].

Generation X participants emphasised physical strain, reduced tolerance for night shifts and the need for predictable schedules. These findings align with literature showing that a higher number of monthly night shifts increases work–family conflict and impacts well‐being [42, 43]. The prominence of logistical stressors—such as parking difficulties and long commutes—among older nurses suggests that retention strategies for this group should address both organisational and infrastructural factors.

Several nurses described the importance of being treated as unique individuals rather than interchangeable staff members. Feeling personally acknowledged and respected by colleagues and supervisors was perceived as a protective factor, whereas the absence of such recognition contributed to emotional detachment and a weakened sense of belonging.

Interpersonal appreciation also played a significant role. Some participants highlighted that the esteem of colleagues and a supportive team environment helped sustain motivation, while a lack of relational recognition reinforced their decision to leave.

The findings also underscore the importance of professional recognition and career development, especially for middle‐aged nurses. Participants perceived limited opportunities for advancement and insufficient access to training due to staffing shortages. This aligns with literature showing that participation in hospital affairs and recognition of competencies are key determinants of retention [44]. The perception that private healthcare offers clearer career pathways further highlights the need for public hospitals to strengthen internal development opportunities. For a subset of younger nurses, instead, resignation was also linked to broader aspirations for personal and educational growth. Some described leaving as an opportunity to pursue further studies or explore professional paths outside nursing, suggesting that for part of the workforce the nursing role is perceived as a transitional stage rather than a long‐term career destination.

By focusing on enacted resignation rather than intention, our findings highlight how these pressures translate into definitive decisions to leave. Notably, participants emphasised factors that are often under‐represented in ITL literature, such as commuting burden, distance from family and logistical barriers. These quality of life determinants—rarely examined in previous studies—played a decisive role in resignation, emerging as significant amplifiers of daily stress, suggesting that organisational retention strategies should extend beyond clinical workload and consider broader life‐context factors.

Overall, the findings suggest that voluntary resignation reflects the interaction between organisational conditions and life‐stage–related needs rather than isolated job dissatisfaction. Retention strategies should therefore adopt a life‐stage‐sensitive approach, addressing workload, wellbeing, leadership, career development and logistical support in ways that align with the evolving priorities of different generational groups.

### 3.1. Study Limitations

A limitation of this study is the absence of audio recordings; detailed field notes were used instead, which may have reduced the richness of verbatim quotations. However, immediately expanded field notes, dual note‐taking and participant confirmation of key points were employed to enhance accuracy and trustworthiness. Another limitation is that the sample included only hospital‐based nurses, which may restrict the transferability of the findings. The presence, during interviews, of a health profession manager may have limited the expressions of participants, but they have already resigned and could be interviewed without being influenced by concerns about judgement or potential consequences.

### 3.2. Implications

The findings of this study offer several actionable implications for nurse managers seeking to strengthen workforce retention across different life stages.

The actions described require collaboration with the human resources department, healthcare administrators and other relevant stakeholders within the specific healthcare context. At the same time, nurse managers have considerable autonomy in addressing and implementing strategies related to several of the themes identified through the thematic analysis.

*Implement structured, life-stage-sensitive onboarding pathways*: Younger nurses reported insufficient mentoring, premature autonomy and unmet expectations regarding ward placement. Nurse managers should ensure structured onboarding programmes that include adequate shadowing, gradual responsibility assignment and regular feedback. Evidence suggests that structured onboarding improves competency and job satisfaction [35]. Tailoring onboarding to the needs of early‐career staff may reduce emotional exhaustion and support professional integration.

*Strengthen leadership presence, communication and relational support*: Across all generations, participants emphasised the importance of supportive leadership, clear communication and emotional availability [33, 34]. Nurse managers should adopt leadership practices that foster psychological safety, encourage open dialogue and promote team cohesion. Particular attention should be given to improving interprofessional collaboration and addressing hierarchical communication patterns that undermine professional autonomy.

*Enhance recognition and career development opportunities*: Midcareer nurses highlighted limited access to training, insufficient recognition of competencies and unclear career pathways. Nurse managers can mitigate these issues by ensuring equitable access to continuing education, recognising advanced skills and developing transparent career progression frameworks. Supporting professional growth may reduce turnover among experienced staff [29, 31, 32, 45].

*Promote flexible and predictable scheduling models*: Work–life imbalance was the primary driver of resignation across all cohorts. Managers should explore scheduling solutions that increase predictability, reduce excessive night duties and accommodate personal or family responsibilities [46]. Flexibility is particularly important for Generation Z nurses balancing work with academic commitments and for Generation X nurses managing caregiving roles.

*Address workload and staffing shortages proactively*: Chronic understaffing contributed to emotional strain, unsafe working conditions and reduced job satisfaction [47]. Nurse managers should advocate for adequate staffing levels, optimise skill mix and monitor workload distribution to ensure safe and sustainable working environments. These findings suggest that age‐sensitive organisational practices, such as adjusting shift patterns or providing ergonomic support, may be particularly beneficial for midcareer and older nurses [48].

*Consider logistical and quality-of-life factors in retention strategies*: Participants identified commuting burden, parking difficulties and housing challenges as significant contributors to resignation. Although these factors extend beyond traditional managerial domains, nurse managers can collaborate with hospital leadership to explore solutions such as improved parking access, transport support, or housing assistance for nonlocal employees.

*Adopt personalised retention strategies that acknowledge individual identity and aspirations*: Several nurses emphasised the importance of being treated as unique individuals and valued members of the team. Others described resignation as part of a broader personal or educational trajectory. Nurse managers should engage in regular, individualised conversations [49] to understand staff aspirations, provide tailored support and recognise personal contributions. Such relational practices may strengthen commitment and reduce turnover.

Overall, these findings suggest that effective retention requires a multifaceted approach that integrates organisational, relational and life‐stage‐specific strategies. By addressing both structural and human factors, nurse managers can create more supportive, sustainable and responsive work environments.

### 3.3. Recommendations for Future Research

Future research should evaluate the effectiveness of retention interventions and extend the investigation to physicians and other nurses, whose professional experience may present distinct patterns.

## 4. Conclusion

This study provides new insights into the reasons underlying voluntary resignation among nurses by focusing on individuals who had already enacted their decision to leave, rather than on intention‐based measures. The findings show that resignation is a multifactorial process shaped by the interaction between organisational conditions and life‐stage–related needs. While workload, staffing shortages and limited managerial support were common across all generations, the specific triggers varied: younger nurses struggled with insufficient onboarding and the need to balance work with academic or personal commitments; midcareer and middle‐aged nurses emphasised organisational climate, recognition and career development; and later‐career nurses highlighted physical strain, scheduling needs and logistical barriers.

The study also identifies under‐recognised determinants of turnover, such as commuting burden, parking difficulties and the desire for personal or educational growth beyond the nursing role. These findings suggest that effective retention strategies must extend beyond traditional workforce measures and incorporate broader quality of life and identity‐related factors.

By addressing organisational, relational and logistical factors in a coordinated manner, nurse managers can create more supportive and sustainable work environments, ultimately reducing turnover and strengthening the stability of the healthcare workforce.

## Funding

This research received no specific grant from any funding agency in the public, commercial, or not‐for‐profit sectors. Open access publishing was facilitated by the Universita degli Studi di Padova, as part of the Wiley‐CRUI‐CARE agreement.

## Conflicts of Interest

The authors declare no conflicts of interest.

## Supporting Information

Additional supporting information can be found online in the Supporting Information section.

## Supporting information


**Supporting Information** Supporting Table 1. Sociodemographic characteristics of participants. Supporting 2. Interview’s questions. Supporting Figure 1. Decision to resign framework.

## Data Availability

The data that support the findings of this study are available on request from the corresponding author. The data are not publicly available due to privacy or ethical restrictions.
