# Supplementary material for: Why Nurses Resign: A Qualitative Study Across Life Stages and Implications for Workforce Management
Source: J Nurs Manag. 2026 Aug 31;2026:3278481. doi: 10.1155/jonm/3278481 (PMC13527864; doi:10.1155/jonm/3278481)
Supplement: Supplementary file 1 [file JONM-2026-3278481-s001.zip › rev cleansupplementary JAN (1).docx]

*Supplementary Table 1- Sociodemographic characteristics of participants*

| ID | Age (years) | Work-experience (years) |
| --- | --- | --- |
| P3 | 27 | 2,5 |
| P6 | 30 | 3,5 |
| P7 | 31 | 5 |
| P8 | 27 | 1 |
| P9 | 28 | 3 |
| P10 | 32 | 6 |
| P11 | 25 | 2 |
| P12 | 24 | 1 |
| P13 | 28 | 1 |
| P14 | 32 | 4 |
| P16 | 43 | 10 |
| P17 | 33 | 4 |
| P18 | 24 | 1 |
| P19 | 22 | 0,2 |
| P21 | 28 | 3 |
| P22 | 31 | 3 |
| P23 | 29 | 4,5 |
| P24 | 36 | 4 |
| P25 | 36 | 11 |
| P27 | 28 | 0,5 |
| P28 | 31 | 5 |
| P29 | 37 | 4,5 |
| P30 | 31 | 0,5 |
| P32 | 47 | 21 |
| P33 | 27 | 3,5 |
| P34 | 51 | 22,5 |
| P35 | 44 | 18 |
| P36 | 42 | 4 |
| P37 | 35 | 7 |
| P38 | 33 | 6,5 |
| P39 | 49 | 27 |
| P40 | 35 | 12 |
| P41 | 58 | 29 |
| P42 | 28 | 3,5 |
| P43 | 29 | 5 |
| P44 | 27 | 38 |
| P46 | 35 | 8 |
| P47 | 32 | 3,5 |
| P48 | 28 | 3 |
| P49 | 22 | 0,2 |
| P51 | 42 | 3 |
| P52 | 26 | 3,5 |
| P54 | 29 | 4 |
| P55 | 24 | 0,5 |
| P56 | 32 | 7 |
| P57 | 34 | 4,5 |
| P58 | 35 | 9,5 |
| P59 | 35 | 3 |
| P60 | 32 | 7 |
| P61 | 58 | 38 |
| P63 | 25 | 3 |
| P64 | 29 | 4 |
| P65 | 27 | 2 |
| P67 | 25 | 1 |
| P69 | 26 | 3 |
| P70 | 28 | 1 |
| P71 | 30 | 5 |
| P72 | 36 | 5 |
| P73 | 41 | 0,4 |
| P75 | 29 | 5 |
| P76 | 25 | 1,5 |
| P78 | 26 | 1 |
| P79 | 27 | 1,5 |
| P81 | 53 | 5 |
| P83 | 27 | 1 |
| P84 | 35 | 5 |
| P86 | 29 | 1 |

*Supplementary 2 Interview’s questions*

a) What is prompting you to resign today, and what triggered this need?
b) Are there any factors that could have influenced your decision to leave the organization and potentially changed it?
c) What suggestions do you have to make the organization more attractive? Do you have any recommendations for improving the work environment or organizational culture?

*Supplementary Figure 1 Decision to resign framework*

Life-stage priorities (family demands, career stage, education, physical tolerance)

Organizational constraints

(workload, staffing, recognition,

leadership, support)

Cumulative strain

(progressive mismatch between

work demands and life needs)

Threshold

(tipping point of unfeasibility)

Enacted resignation

(voluntary decision to leave)
