# Supplementary material for: Why Nurses Resign: A Qualitative Study Across Life Stages and Implications for Workforce Management
Source: J Nurs Manag. 2026 Aug 31;2026:3278481. doi: 10.1155/jonm/3278481 (PMC13527864; doi:10.1155/jonm/3278481)
Supplement: Supplementary file 1 [file JONM-2026-3278481-s001.zip › rev cleanTable_coding_tree_complete 2.docx]

*Table 2 – Description of the coding tree*

| THEMES | SUB-THEMES | CODES | QUOTATIONS |
| --- | --- | --- | --- |
| Negotiating life priorities and work–life misalignment | Personal and financial drivers of relocation | Moving closer to home | “I need to move closer to my family.” (P8) |
|  |  | High rental costs | “Rents here are too high for my salary.” (P33) |
|  | Work-life incompatibility | Study–work conflict | “Work cannot be reconciled with my study commitments.” (P48) |
|  |  | Early emotional exhaustion | “I’m already emotionally drained.”(P70) |
| Early-career transition challenges and unmet support needs | Lack of organisational support | Insufficient onboarding | “I was given autonomy too quickly, without adequate guidance.” (P44) |
|  |  | Feeling abandoned | “They left me alone with all the patients with no feedback” (P61) |
|  |  | Premature autonomy | “They gave me autonomy very quickly.” (P44) |
|  | Unsustainable workload intensity | Excessive workload | “The workload is huge and responsibilities are very high.” (P83) |
|  |  | Staff shortages | “We are always understaffed.” (P18) |
| Experiencing work–life strain within challenging workplace environments | Personal life incompatibility | Work–family conflict | “Work is not compatible with my family situation.” (P62) |
|  |  | Excessive workload and Chronic fatigue | “The workload is huge… I don’t want to be treated like a number.” (P84) “I’m exhausted; emotional stress is affecting my health.” (P51) |
|  | Dysfunctional workplace relationships | Conflicts | “This is a toxic environment.” (P29) |
|  |  | Lack of listening | “Nobody really listens to our problems.” (P16) |
| Experiencing limited recognition and constrained professional growth | Blocked professional development | No continuing professional development | “I asked several times for courses, but they were denied.” (P46) |
|  |  | Desire for internal mobility | “I would have liked to experience other wards.” (P84) |
|  | Lack of professional recognition | Skills not valued | “I was never formally recognised for my advanced skills.” (P51) |
|  |  | Unrecognised competencies | “I had many responsibilities, but no recognition.” (P59) |
| Accumulating physical strain and everyday workplace burdens | Physical Demands and Age-Related Fatigue | Chronic fatigue | “Physically I can’t do it anymore.” (P81) |
|  |  | Reduced recovery capacity | “At my age, recovery after a night shift is difficult.” (P39) |
|  | Cumulative Stress Exposure | Parking difficulties | “I can’t find parking.” (P81) |
|  |  | Emotional exhaustion | “I’m 59 and exhausted… stress has built up over time.” (P41) |
| Negotiating Work–Life Boundaries | Caregiving as a Catalyst for Resignation | Need for more personal time | “I need to work part-time to manage my family responsibilities.” (P34) |
|  | Reorientation Toward Sustainable Work | Denied part-time | “I asked for part-time but it was never granted.” (P61) |
|  |  | Preference for day shifts | “Day shifts are the only sustainable option for me.” (P34) |
